# The effect of Biokos™, a natural lipopeptide surfactant extracted from the bacterium *Pseudomonas*, on *Epistylis* infections in *Carassius auratus*

**DOI:** 10.1590/S1984-29612024047

**Published:** 2024-08-21

**Authors:** Gabriela Cavalante Maciel, Simone de Carvalho Balian, Herbert Sousa Soares, Maurício Laterça Martins, Giovanni Salerno, Malte Jarlgaard Hansen, Pedro Henrique Magalhães Cardoso

**Affiliations:** 1 Curso de Medicina Veterinária, Universidade Anhembi Morumbi – UAM, São Paulo, SP, Brasil; 2 Departamento de Medicina Veterinária Preventiva e Saúde Animal, Faculdade de Medicina Veterinária, Universidade de São Paulo – USP, São Paulo, SP, Brasil; 3 Programa de Pós-graduação em Saúde Única, Universidade Santo Amaro, São Paulo, SP, Brasil; 4 Laboratório de Sanidade de Organismos Aquáticos – AQUOS, Departamento de Aquicultura, Universidade Federal de Santa Catarina – UFSC, Florianópolis, SC, Brasil; 5 Sundew ApS, Copenhagen, Denmark

**Keywords:** Aquatic health, parasite, ciliated protozoan, lipopeptide, ornamental fish, Saúde aquática, parasita, protozoário ciliado, lipopeptídeo, peixes ornamentais

## Abstract

In the aquaculture industry, biocides are routinely used to treat parasitosis in fish, and researchers are continually developing sustainable alternatives that can replace these harsh chemicals. In this context, the objective of this study was to evaluate the effectiveness of a new natural compound, Biokos^TM^, for the treatment against *Epistylis* sp. in *Carassius auratus* fish. The infestation was identified by the presence of whitish plaques on the integument of five animals, and the diagnosis was confirmed through skin scrapings. Biokos^TM^ is a lipopeptide derived from the bacteria *Pseudomonas* that can destroy the functionality of the cell membrane of ciliated protozoa. The action of Biokos^TM^ does not harm animals and the environment because the compound degrades into amino acids and fatty acids within days. A 0.15 m^3^ (150 L) aquarium was treated with an Ich-Away^TM^ water conditioner manufactured by the Danish company Sundew ApS, which has Biokos^TM^ as the active ingredient. Six tablets were added to the water daily for two days, and new skin scrapings were performed. The fish were clinically well and no longer possessed lesions or parasites. The results obtained indicate that Biokos^TM^ can be an innovative and more sustainable alternative for controlling epistyliasis in ornamental fish.

## Resumo

Substâncias químicas, potencialmente lesivas, são rotineiramente utilizadas no tratamento de parasitoses na indústria da aquicultura. Alternativas sustentáveis são constantemente buscadas para a substituição desses métodos. Nesse contexto, o objetivo deste estudo foi avaliar a eficácia de um novo composto de origem natural chamado Biokos^TM^, no tratamento contra *Epistylis* sp. em *Carassius auratus*. A infestação foi identificada pela presença de placas esbranquiçadas no tegumento de cinco animais e o diagnóstico confirmado por raspados de pele. Biokos^TM^ é um lipopeptídeo derivado da bactéria *Pseudomonas,* capaz de destruir a funcionalidade da membrana celular de protozoários ciliados. Sua ação é pouco agressiva aos animais e ao meio ambiente, degradando-se em aminoácidos e ácidos graxos em poucos dias. O aquário de 0.15 m^3^ (150 L) foi tratado com o condicionador de água Ich-Away^TM^ – fabricado pela empresa dinamarquesa Sundew ApS – que possui o Biokos^TM^ como princípio ativo. Foram utilizados seis tabletes por dia, durante dois dias. Após o tratamento foram realizados novos raspados de pele, que não apresentaram mais lesões ou parasitos e os peixes se apresentavam clinicamente bem. Os resultados obtidos indicam que o Biokos^TM^ pode ser uma alternativa inovadora e sustentável no controle da epistilíase em peixes ornamentais.

## Introduction

The ornamental freshwater fish industry is an important part of the companion animal market, and the ornamental fish trade has been growing annually in Brazil; these fish are the fourth most prevalent category of pets in households, and in 2022, there were approximately 22.2 million ornamental fish in Brazil (Abinpet, 2023). Brazil is among the main suppliers of ornamental species from tropical climates, the majority of which are captured; and this is also the second largest exporter of these fish in South America and the 17th largest exporter in the world (Portz et al., 2013).

The ornamental goldfish (*Carassius auratus*), popularly known as Kinguio in Brazil, is very popular among aquarium hobbyists. Fish of this species generally have long, oval-shaped bodies, and the shape of their tails varies depending on the mutation. The color of the fish ranges from golden brown to white, black, red, orange, yellow, gray or even combinations of these colors. The fish also exhibit great polymorphism, which can determine different shapes of fins, eyes and other parts of the body, as well as their color (Froese & Pauly, 2024). Goldfish are among the most common fish bred by aquarists and are a popular choice due to their beauty and ease of handling (Wildgoose, 2001). Goldfish have a life expectancy of 10 years in aquariums but can live for up to 30 years in lakes (Kunii, 2010).

The intensification of aquaculture is among the factors responsible for the increase in the occurrence of parasitic diseases in aquatic organisms, especially fish; intensive animal breeding systems, transport and trade create ideal conditions for the occurrence and spread of parasitic diseases due to imbalances among the host, environment and pathogenic agents. Animals subjected to stressful situations, which are often inherent to this system, are more susceptible to the action of parasites and other pathogens. An imbalance in water quality parameters and a lack of biosafety measures in facilities, in addition to inadequate handling, increase the risk of infections by bacteria, virus, aquatic fungi and parasite infestations. Preventative measures that guarantee the health of animals must be adopted to prevent high mortality rates and, consequently, economic losses (Dominguez et al., 2023).

The ornamental fish trade requires trained professionals who work in animal handling, pathology, diagnosis and treatment. Despite being a well-established industry, there is a lack of trained professionals with knowledge of adequate handling and sanitary measures in the Brazilian market, so sick fish are often sold to the buyers in the marketing chain (Cardoso et al., 2017; 2019; Dominguez et al., 2023).

Parasites of the genus *Epistylis* are colonial, sessile ciliated protozoa belonging to the order *Peritrichia* that parasitize the integument, fins and gills of fish and cause epistyliasis (Campos et al., 2014). These ectocommensal parasites use the surface of fish to attach themselves and feed on particles suspended in the water; their zooid cells are bell-shaped, with cilia in the apical portion, in addition to a long, branched, noncontractile peduncle colonized by bacteria (Assane et al., 2022). The parasite is not selective for its host but uses the surface of fish as an adhesion substrate (Portz et al., 2013). Scaleless fish (pimelodids) have a lower susceptibility to infestation (Assane et al., 2022). *Epistylis* sp. seeks firm surfaces for attachment, such fin rays, the edges of the operculum, the lips and the surface of the head (Campos et al., 2014). Infestation by *Epistylis* sp. is closely related to the low quality of water and the presence of organic pollution since the parasite feeds on organic particles and bacteria in suspension.

The main clinical sign of infestation by *Epistylis* sp. is the presence of colonies visible to the naked eye with a whitish or yellowish color; the presence of bacteria in the peduncles of the parasite, mostly gram-negative bacilli, is a highly relevant factor. The protozoan and the bacterium *Aeromonas hydrophila* form a symbiotic complex that triggers the pathology known as “Red Sore Disease”, which causes white or hemorrhagic lesions on the flanks or ends of bony prominences of fish (Noga, 2010). Histopathological analysis associated the parasite with hyperplasia of the injured epithelium, hydropic degeneration with multifocal necrosis, proliferation of mucolytic cells, mast cell infiltration and granulocytosis with the presence of giant cells (Assane et al., 2022).

*Epistylis* sp. can reproduce sexually, in which nonsessile stages are formed, or asexually, in which binary division occurs; however, both forms of reproduction are efficient (Campos et al., 2014). In addition to the presence of colonies in the animals, which are visible to the naked eye, infestation by *Epistylis* sp. is diagnosed by microscopy through the identification of zooids with peduncles, which are not contractile; other peritrichia that are organized in colonies, such as *Vorticella, Zoothamnium* and *Carcnesium,* have contractile peduncles (Noga, 2010). Wet mount preparations are diagnostic.

According to Noga (2010), epistyliasis can be treated with baths and prolonged immersions of potassium permanganate, formalin and salt. Each of these therapeutic agents has specific methods of use, which consider the dosage and exposure time of the animals as well as water parameters, such as temperature and pH, particularly in the case of formalin and potassium permanganate. Currently, few publications have explored possible treatments for epistyliasis in farmed and ornamental fish.

Conventional treatments used to treat free-living protozoa in aquatic environments can be toxic to the host and environment (Sudova et al., 2008). Chemicals such as malachite green, copper sulfate, methylene blue and formalin are widely used in industry for their antiparasitic benefits; however, these chemicals also pose risks to those handling the animals as well as the fish being treated. These agents are environmental pollutants that may become a risk to nontarget organisms, such as plants and invertebrates (Lieke et al., 2019).

Formalin, a liquid formaldehyde solution at a standard concentration of 37%-40%, has been widely used in industry to control various parasitic diseases, particularly those of the skin, fins and gills. However, formalin poses risks to handlers—since the solution is highly irritant and a potential carcinogen—and to the fish being treated (Tavares-Dias, 2021). Fish can be subjected to short or prolonged formalin baths, depending on the tolerance and condition of the animals, water quality and parasite species; factors such as pH and water temperature can increase the toxicity of this chemical and should be considered prior to administration. Behavior alterations in fish exposed to this chemotherapeutic agent may include jumping due to skin irritation, agitation, respiratory distress, loss of balance, erratic swimming, lethargy, exophthalmia, crowding on the water surface, loss of hydrodynamic equilibrium, spasms, agonistic confrontation, darkening of the body, sudden and quick movement and excessive accumulation of mucus. These problems are related to a decrease in oxygen consumption, possibly caused by to the gill epithelium; exposure to formalin also causes damage to liver, kidney and spleen (Tavares-Dias, 2021).

Malachite green, a basic dye soluble in water, is known to provide effective treatment against protozoan ectoparasites. Its use is restricted to aquarium and ornamental fish breeding since it persists in edible fish tissues for extended periods of time. For that reason, it was banned in the EU in 2000; tests carried out on warm-blooded animals demonstrated the carcinogenicity and teratogenicity of malachite green and it also causes eye irritation in humans. Malachite green is used in dips, short-term and long-term baths to treat fungal infections and parasite infestations. Despite its efficacy, this chemical is highly toxic to fish since lethal concentrations and therapeutic concentrations are sometimes very close to each other; fish can become rapidly intoxicated and show symptoms such as restlessness and uncoordinated movements. Animals also present loss of balance, apathy and agony, prior to death. The gills become edematous, discolored and there is excessive mucous matter; the skin presents a green tinge and excessive production of slime. There is evidence of vessel dilation in the body cavity and the organs become light green in colour (Sudova et al., 2008).

Methylene blue is another known dye used in aquaculture. According to Noga (2010), it has been advocated in aquarium literature as a treatment for ectoparasite infestations and nitrite toxicity by prolonged immersion; however other chemicals have stronger evidence of efficacy. Although many over-the-counter pharmaceuticals contain this ingredient, its use is not recommended in systems with biological filtration as it is toxic to nitrifying bacteria. It should be handled with caution as it stains objects (Noga, 2010). Like malachite green, methylene blue is known to persist in tissue for extended periods of time, posing a risk not only to animals but also to humans when fish are consumed; it is labeled as toxic by EU regulation and deleterious to human health and the environment. Highly concentrated methylene blue has been linked to neurotoxicity and encephalopathy (Lieke et al., 2019).

Copper sulfate is another largely used chemical in aquaculture for its anti-parasitic properties and its applications involve protozoan and monogenoidean control. However, studies show that excess copper may alter blood parameters and osmoregulation of fish as it damages gill epithelium, hematopoietic tissues, liver, kidney and spleen of animals; immunosuppression is also observed as monocytes and neutrophils are sensitive to heavy metals. Copper sulfate may accumulate on fish tissues so animals cannot be consumed; its toxicity is influenced by alkalinity and hardness, demanding extra caution from handlers when used (Tavares-Dias et al., 2011).

Biokos™, a cyclic lipopeptide, is a surfactant derived from a naturally occurring *Pseudomonas* bacterium found in fish farm environments; it has emerged as an innovative solution for controlling aquatic parasites (Liu et al., 2015). Biokos™ can eliminate various protozoan fish parasites and has specifically shown significant inhibitory effects on ciliate parasites such as *Ambiphyra* sp., *Chilodonella* sp., C*ryptocaryon* sp., *Ichthyophthirius multifiliis*, *Tetrahymena* sp. and *Trichodina* sp., and also against some non-ciliate parasites, e.g., *Ichthyobodo* sp. (Al-Jubury et al., 2018; Li et al., 2022; Marana et al., 2023; Watanabe et al., 2023). The lipopeptide is environmentally benign, non-persistent in water as it degrades into amino acids and fatty acids within days and is not harmful to biofilter bacteria, which have the crucial role in removing nitrogenous waste products from fish rearing and fish keeping systems (Hansen et al., 2022). Additionally, compared with conventional products, Biokos™ is safer to handle (formalin, malachite green, etc.). It is formulated as a predosed effervescent tablet. Due to these properties, Biokos™ is a viable and promising solution for combatting protozoan parasites in both aquaculture and ornamental fish. This work aimed to explore this novel compound as an alternative treatment for epistyliasis in ornamental goldfish in Brazil.

## Case Report

For this case, 28 *C. auratus* with an average weight of 30 g obtained from an ornamental fish distributor located in greater São Paulo, southeastern Brazil, were analyzed. Originally, the fish were grown by the breeder in tanks and, once at the distributor, stored in 0.15 m^3^ (150 L) aquariums in a closed system with a foam filter and fed on average twice a day with Dr. Bassleer^®^ commercial granulated food. Water quality parameters were measured weekly and maintained, on average, at the following values: temperature 26 °C, salinity 1000 ppt, pH 7.5, total ammonia 0.0 mg L^-1^ and nitrite 0.0 mg L^-1^.

**Figure 1:**
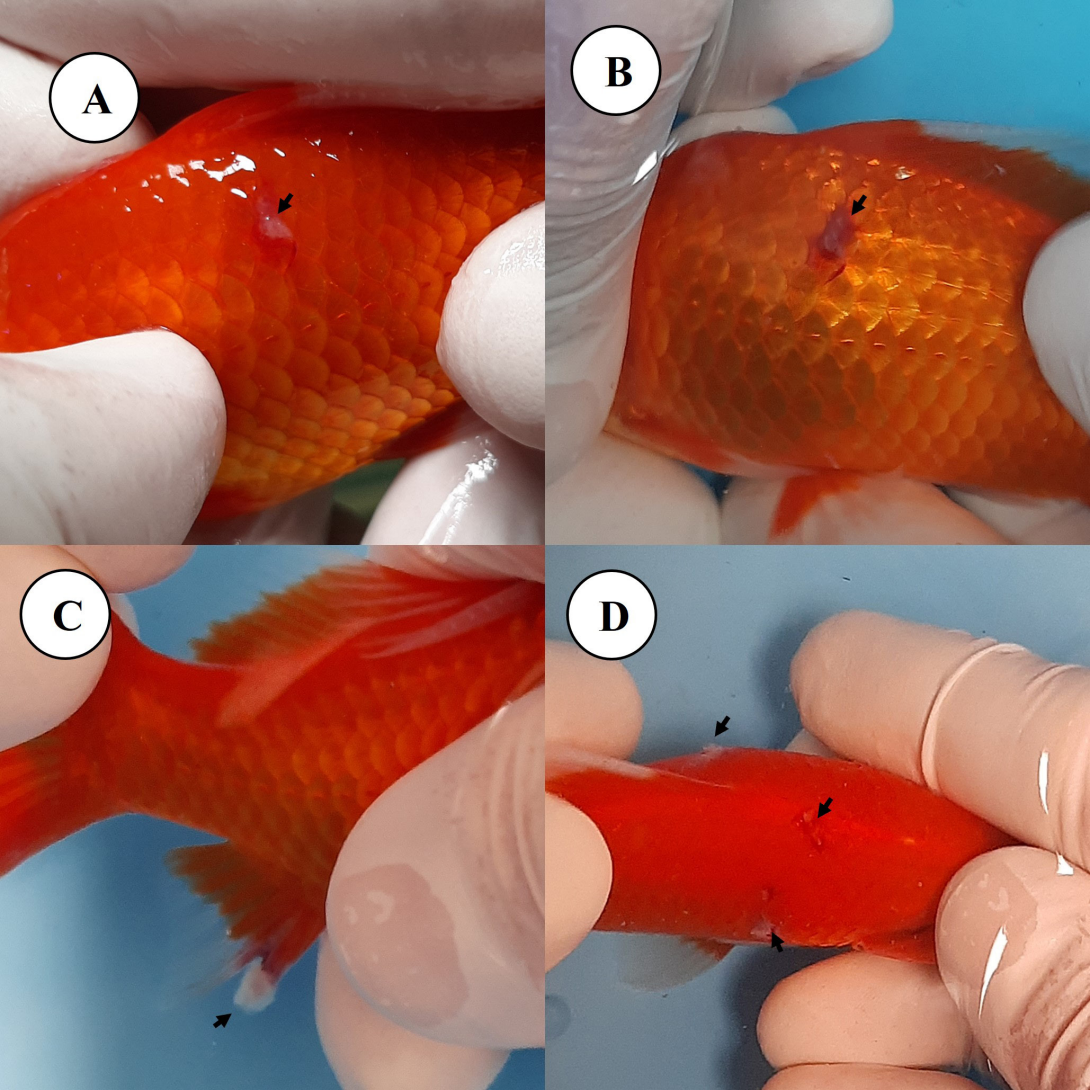
Goldfish (*Carassius auratus*) with white plates on the left side of the body (A-B), on the anal fin (C) and on the skin on the left, right and dorsal sides of the body (D).

**Figure 2:**
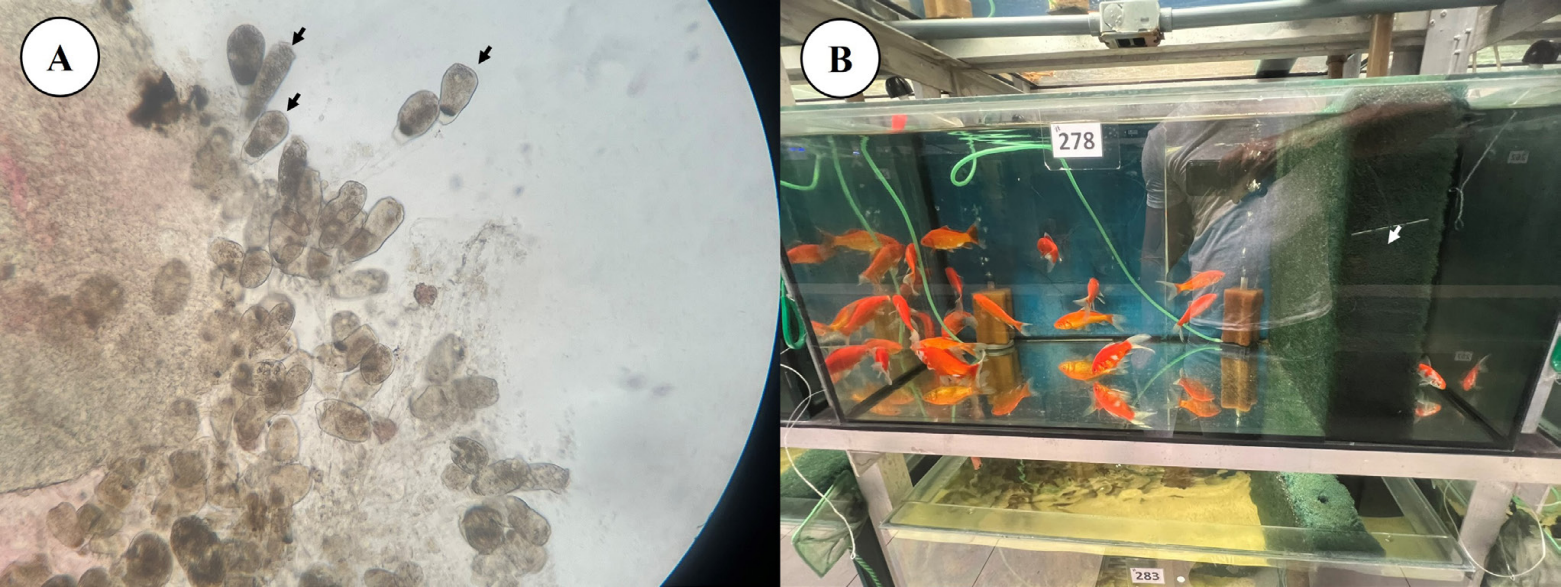
*Epistylis* on wet mount preparations under a light microscope at 20x magnification (A) and all 28 fish treated in the same aquarium (B) separated by the general population by a foam (white arrow).

During acclimation at the distributor, small plaques (Figure 1A-D) with a velvety appearance and a whitish color were observed on the skin and fin, especially the dorsal and caudal ones, and on the opercula of several fish. Five animals were subjected to skin scraping of the affected areas, and the parasite *Epistylis* sp. was identified through analysis of wet mount preparations under a light microscope using 4x, 10x and 20x objectives (Figure 2A). These fish were placed in the same aquarium as the others but were separated from the general population by a foam (Figure 2B - white arrow) wall for monitoring.The selected treatment protocol involved the use of an Ich-Away^TM^ water conditioner. This product contains the active ingredient Biokos^TM^. Three Biokos^TM^ tablets (1125 mg) is recommended by the manufacturer to treat .15 m^3^ (150 L) of water, or 7.5 mg L^-1^. The dose chosen by veterinarian against *Epistylis*, which was not in the Biokos^TM^ leaflet, was double recommended by the manufacturer, that is, 15 mg L^-1^, because this parasite is difficult to control with the most common available drugs, so six effervescent tablets were used per day for two days, with each tablet containing 375 mg of Biokos^TM^. On the second day after use, the five symptomatic fish selected at the beginning of treatment were subjected to new skin scraping, and all tested negative for *Epistylis* sp. The other fish in the aquarium were visually assessed, and it was verified that none of the animals displayed whitish plaques characteristic of infestation by the parasite, highlighting the success of the chosen treatment protocol.

## Discussion

Several methods have been used by industry to treat epistyliasis, with varying effectiveness and limitations depending on the type of facility and destination of the animals. The use of certain substances to treat parasites in fish is limited, especially in the case of farmed animals, as these substances accumulate in the tissues of these fish and exert their deleterious effects on humans (Sudova et al., 2008). The treatment of diseases in ornamental fish is less restricted since these animals are not intended for human consumption. Thus, alternate solutions are needed that provide effective treatments against parasites in farmed and ornamental fish without the possible hazards related to these chemicals. Biokos^TM^ has emerged as an alternative compound for the treatment of protozoan parasites in the ornamental fish industry as opposed to traditional treatment methods.

According to the company Sundew that produces Biokos^TM^, its use is intended to eliminate the parasites *Ambiphyra* sp., *Chilodonella* sp., *Ichthyobodo* sp., *Ichthyophthirius multifiliis, Philasterides, Tetrahymena* sp. and *Trichodina* sp., while its antiparasitic efficacy on the protozoan *Epistylis* sp. is not known. The chosen concentration, 15 mg L^-1^ of Biokos^TM^, was effective in eliminating the protozoa without causing harmful effects on the fish; during and after treatment, the fish appeared alert and showed no signs of stress. There were no loss or adverse effects in terms of appetite, integument color, swimming pattern, breathing or general appearance of the animals. In summary, the fish were not negatively affected by the treatment at the dose administered.

After treatment, the fish subjected to skin scraping tested negative for *Epistylis* sp.; moreover, no other protozoa were found to parasitize the animals’ integument. The fish in the treated aquarium were monitored for fourteen days after the end of treatment, and there was no recurrence of epistyliasis.

The parasites were effectively eliminated without the need to subject the animals to treatments with aggressive therapeutic agents, as is customary. Furthermore, treatment with Biokos^TM^ was be simpler and safer than traditional options in terms of handling and exposing the technical team to chemical compounds harmful to humans.

Research into natural treatment methods in aquaculture has been strongly encouraged by the industry and scientific community, which are constantly aiming to find green therapeutic agents that can replace antibiotics, disinfectants and highly toxic chemicals for the treatment of animals. Biokos is degraded after 1-2 days, slower in more sterile environments, and faster in more mature ones as it depends on bacterial activity. The degradation of Biokos has been assessed by Korbut et al. (2022) which found an average decrease of >70% in the Biokos concentration after 24 h at the therapeutic doses (5-20 mg active compound/L).

In this context, we concluded that Biokos^TM^ is a promising and suitable option for treating *Epistylis* sp. and possibly other protozoan parasites, and one of its main attributes is its biodegradability. We believe that more detailed research is necessary to enrich this initial study in Brazil. In addition, this study can serve as a basis for new research that has a more adjusted design.

## References

[B001] Al-Jubury A, Lu C, Kania PW, von Gersdorff Jørgensen L, Liu Y, de Bruijn I (2018). Impact of *Pseudomonas* H6 surfactant on all external life cycle stages of the fish parasitic ciliate *Ichthyophthirius multifiliis.*. J Fish Dis.

[B002] Assane IM, Moreira LF, Gallani SU (2022). Parasitoses causadas por protozoários ciliados. Cad Téc Vet Zootec.

[B003] ABINPET (2023). Mercado Pet Brasil.

[B004] Campos CM, Rodrigues RA, Oliveira CAL, Nunes AL, Fantini LE, Ushizima TT (2014). Permanganato de potássio como agente terapêutico no controle de *Epistylis* sp. em cachara *Pseudoplatystoma reticulatum* e seus efeitos na hematologia. Bol Inst Pesca.

[B005] Cardoso PHM, Balian SC, Matushima ER, Pádua SB, Martins ML (2017). First report of scuticociliatosis caused by *Uronema* sp. in ornamental reef fish imported into Brazil. Rev Bras Parasitol Vet.

[B006] Cardoso PHM, Balian SC, Soares HS, Tancredo KR, Martins ML (2019). *Neobenedenia melleni* (Monogenea: Capsalidae) in ornamental reef fish imported to Brazil. Rev Bras Parasitol Vet.

[B007] Dominguez HN, Balian SC, Relvas RS, Soares HS, Queiroz MR, Martins ML (2023). Parasitological diagnosis in ornamental freshwater fish from different fish farmers of five Brazilian states. Braz J Biol.

[B008] Froese R, Pauly D (2024). Carassius auratus (Linnaeus, 1758). Goldfish.

[B009] Hansen MJ, Mehrdana F, Hansen EH, Salerno G, Hansen J (2022). Remedy for combating ich in finfish aquaculture. World Aquac.

[B010] Korbut R, Skjolding LM, Mathiessen H, Jaafar R, Li X, von Gersdorff Jørgensen L (2022). Toxicity of the antiparasitic lipopeptide biosurfactant SPH6 to green algae, cyanobacteria, crustaceans and zebrafish. Aquat Toxicol.

[B011] Kunii EMF (2010). Frequência alimentar e taxa de alimentação para Kinguio criado em hapa: desempenho produtivo e avaliação econômica.

[B012] Li X, Jaafar R, He Y, Wu B, Kania P, Buchmann K (2022). Effects of a *Pseudomonas* H6 surfactant on rainbow trout and *Ichthyophthirius multifiliis: in vivo* exposure. Aquaculture.

[B013] Lieke T, Meinelt T, Hoseinifar SH, Pan B, Straus DL, Steinberg CEW (2019). Sustainable aquaculture requires environmental-friendly treatment strategies for fish diseases. Rev Aquacult.

[B014] Liu Y, Rzeszutek E, van der Voort M, Wu CH, Thoen E, Skaar I (2015). Diversity of aquatic *Pseudomonas* Species and their activity against the fish pathogenic oomycete saprolegnia. PLoS One.

[B015] Marana MH, Al-Jubury A, Mathiessen H, Buchmann K (2023). Lipopeptide surfactant killing of *Ichthyophthirius multifiliis*: mode of action. Aquacult Rep.

[B016] Noga EJ (2010). Fish disease: diagnosis and treatment..

[B017] Portz L, Antonucci AM, Ueda B, Dotta G, Guidelli G, Roumbedakis K, Pavanelli GC, Takemoto RM, Eiras JC (2013). Parasitologia de peixes de água doce do Brasil..

[B018] Sudova E, Machova J, Svobodova Z, Vesely T (2008). Negative effects of malachite green and possibilities of its replacement in the treatment of fish eggs and fish: a review. Vet Med (Praha).

[B019] Tavares-Dias M, Ferreira JS, Affonso EG, Ono EA, Martins ML (2011). Toxicity and effects of copper sulfate on parasitic control and hematological response of tambaqui *Colossoma macropomum.*. Bol Inst Pesca.

[B020] Tavares-Dias M (2021). Toxicity, physiological, histopathological and antiparasitic effects of the formalin, a chemotherapeutic of fish aquaculture. Aquacult Res.

[B021] Watanabe Y, Hansen J, Kotake M, Fujii R, Matsuoka H, Yoshinaga T (2023). Effect of Biokos, a natural lipopeptide surfactant extracted from a bacterium of the *Pseudomonas* genus, on infection of *Cryptocaryon irritans.*. J Fish Dis.

[B022] Wildgoose WH (2001). BSAVA manual of ornamental fish..

